# Multi-Color Tunable Afterglow Materials Leveraging Energy Transfer Between Host and Guest

**DOI:** 10.3390/molecules30061203

**Published:** 2025-03-07

**Authors:** Xiao He, Bo Wang, Xiaoqiang Zhao, Fengqin Ke, Wenhui Feng, Liwen Wang, Jiameng Yang, Guangyu Wen, Denghui Ji

**Affiliations:** 1 Department of Thermal Engineering, Hebei Petroleum University of Technology, Chengde 067000, China; hexiaocdpc@163.com (X.H.); 13832454905@163.com (F.K.); wenhuicuihua@126.com (W.F.); liwen00918@163.com (L.W.); m17531089917@163.com (J.Y.); 2 Hebei Advanced Thin Film Laboratory, College of Physics, Hebei Normal University, Shijiazhuang 050024, China; gywen@hebtu.edu.cn; 3 Science College, Shijiazhuang University, Shijiazhuang 050035, China

**Keywords:** multi-color tunable afterglow materials, phosphorescent material, Förster energy transfer

## Abstract

Host/guest doping is an effective approach to achieving room-temperature phosphorescence (RTP). However, the influence of the host matrix on doping systems is still unclear, and it is difficult to select the suitable host species for a certain guest emitter. This study prepared a series of host/guest RTP materials with dynamically adjustable time and color by doping a non-RTP guest material in various host materials that were easy to crystallize. The varying afterglow color originated from the difference in Förster energy transfer between the host and guest. Specifically, the change from yellow to green afterglow was realized by varying the host’s molecular structure. This study further revealed the importance of proper host energy levels, the ability to generate long-aging triplet excitons, and the Förster energy transfer from host to guest. Additionally, multiple information encryption anti-counterfeiting materials were developed by leveraging the different afterglow colors and durations, reflecting the unique performance advantages of the prepared long-afterglow materials in various RTP applications.

## 1. Introduction

Due to their long-life luminescence, ease of preparation and modification, and good biocompatibility, organic room-temperature phosphorescence (RTP) materials have been applied in fields such as information encryption, optical sensing, and bioimaging [1,2,3,4,5]. However, organic small molecules are affected by spin-forbidden transitions and weak spin–orbit coupling (SOC) constants, which lowers the rate of intersystem crossing (ISC) between the singlet and triplet systems [6,7,8,9,10]. Moreover, factors such as non-radiative transitions impede the preparation of long-lifetime RTP materials. Researchers have developed various methods to obtain organic materials with efficient and ultra-long phosphorescence, yielding configurations such as small-molecule crystals [11], self-doping [12], hydrogen-bonded organic frameworks (HOFs) [13,14], host/guest doping materials [15,16,17,18,19], and polymer matrices [20,21,22,23]. These approaches can stabilize triplet excitons in ambient conditions to realize high-performance persistent RTP properties [24,25,26]. Despite the recent progress, systematic research and exploration for RTP materials’ lifetime and color-tuning properties are still lacking, inevitably hindering their development and practical application.

Among the reported strategies, crystals have shown great potential in preparing organic long-lifetime afterglow materials [27,28,29,30]. Organic small-molecule crystals, on the other hand, can realize rigid environments that inhibit non-radiative transitions and reduce external environmental influences, suppressing triplet exciton quenching by water and oxygen [31,32]. These properties make organic small molecules a promising method to obtain efficient afterglow materials, although realizing afterglow for pure organic small molecules is still difficult. Host/guest doping facilitates electron charge transfer and promotes the formation of long-life charge separation states, which is an effective approach to achieving afterglow materials with a long lifetime. By adjusting the energy gap between the guest emitter’s lowest singlet and the host matrix’s lowest triplet state, we can alter the ISC process between them to realize a wide-range time- and color-tunable afterglow at room temperature [15,19]. A series of rigid host matrices require proper triplet energy levels between the lowest singlet and triplet states of the guest emitter, along with the ability to isolate guest molecules and promote the energy transfer process. However, the influence of the host matrix on doping systems is still unclear, and it is difficult to select the suitable host species for a certain guest emitter. These problems have restrained the development of host/guest doping materials.

**Figure 1:**
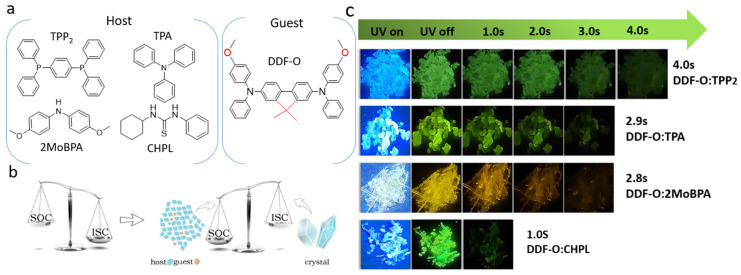
(**a**) Chemical structures of host and guest. (**b**) Mechanism diagram of host/guest-doped material. (**c**) The doped crystals with LPL durations of 1~4 s were prepared by solvent volatilization of the guest-doped host powder followed by crystallization at room temperature (excitation: 365 nm).

In this paper, 2,7-di-(N,N-diphenylamino)-9,9-dimethyl-9H-fluorene (DDF-O) was used as the guest to construct a multi-host/guest system. A series of host molecules with simple structures were screened (Figure 1a), and a simple solution evaporation method was chosen to realize effective host/guest doping and induce long-afterglow properties of host molecules with different structures. By energy transfer of the host/guest molecules, the doped crystals of 4,4′-dimethoxybenzophenone (2MoBPA): DDF-O and triphenylphosphine (TPP_2_): DDF-O showed different afterglow colors. As shown in Figure 1c, after being excited by a 365 nm ultraviolet (UV) source, TPP_2_: DDF-O exhibited a green afterglow of up to 4 s, and 2MoBPA: DDF-O displayed a yellow afterglow of up to 2 s, as proven by their emission decay curves. Specifically, by doping non-RTP guest materials into easily crystallizable host materials, this work achieves a transition from yellow to green afterglow colors, revealing the Förster energy transfer mechanism between the host and guest. Furthermore, the importance of proper energy levels of the host molecules, the ability to generate long-lived triplet excitons, and the energy transfer from the host to the guest for RTP performance are explored. Through this strategy, a concise and effective screening mechanism to develop afterglow materials with different colors and lifetimes was realized. This host/guest doping method can realize wide-range tunable lifetimes and afterglow colors and promises widespread application, such as time-dependent information display, high-level security protection, and dynamic multi-dimensional anti-counterfeiting.

## 2. Results and Discussion

DDF-O was selected as the guest in this study because of its suitable energy level and compatibility. Pure DDF-O does not exhibit phosphorescence at room temperature: the twisted structure of the fluorene group in DDF-O prevents it from easily embedding into flat and compact host matrices. To address this, we selected a series of host matrices that are compatible with the twisted conformation of DDF-O. These hosts included 2MoBPA, TPP, TPA, and CHPL. These hosts can crystallize easily to provide rigid environments and possess distorted molecular structures, facilitating effective guest molecule doping. Strong compatibility between the host and guest molecules is also essential. The ideal host molecule requires a large singlet–triplet energy gap to align with the triplet energy levels of various guest matrices.

**Figure 2:**
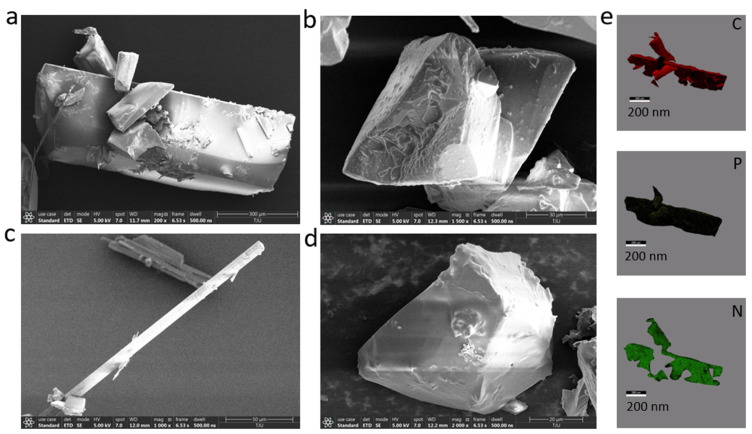
SEM images (**a**) TPP_2_: DDF-O, (**b**) 2MoBPA: DDF-O, (**c**) TPA:DDF-O, and (**d**) CHPL: DDF-O and corresponding EDX mappings of the TPP2: DDF-O crystals. (**e**) corresponding EDX mappings (C, P, N) of the TPP_2_: DDF-O crystals.

Figure S1 includes the XRD pattern of the doped crystal material. We found that the peak positions of the materials were in the range of 10–50°. TPP_2_: DDF-O, 2MoBPA: DDF-O, TPA:DDF-O, and CHPL: DDF-O all exhibited sharp diffraction peaks and flat baselines, indicative of good crystallinity and highly ordered arrangement. However, the peak positions of the material (TPP_2_: DDF-O, 2MoBPA: DDF-O, TPA: DDF-O, and CHPL: DDF-O) are different, which is related to the crystal type. To obtain the elemental distribution information of the doped crystals, we employed surface morphology analysis tools, scanning electron microscopy (SEM), and EDX mapping (Figure 2). As expected, C, P, and N atoms were detected, especially the N atom resulting from the DDF-O molecule. These elements were homogeneously distributed throughout the crystals, also reflecting that the trace amounts of DDF-O added were distributed evenly within the TPP_2_ framework.

**Figure 3:**
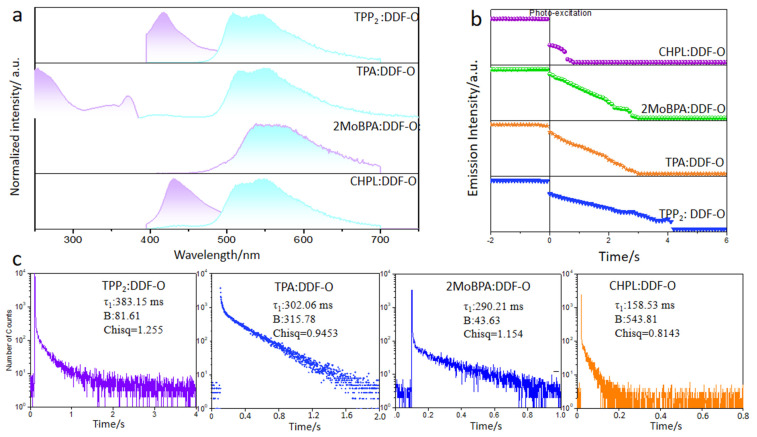
Fluorescence (purple) and phosphorescence (cyan) emission spectra (**a**), semi-logarithmic plot of the emission decay profiles (**b**), and phosphorescence emission decay spectra (**c**) of TPP_2_: DDF-O, 2MoBPA:DDF-O, TPA: DDF-O, and CHPL: DDF-O.

The UV–visible absorption, fluorescence, phosphorescence, and long-afterglow emission spectra of the four types of doped crystals were tested, and the spectral characteristics are shown in Figure 3. The maximum phosphorescence emission wavelength of DDF-O (measured at 77 K) was 520 nm. When the 365 nm UV irradiation was terminated, a bright green afterglow was observed for TPP_2_: DDF-O, TPA: DDF-O, and CHPL: DDF-O, whereas a yellow afterglow was witnessed for 2MoBPA: DDF-O. However, the decay spectra of all the doped crystals showed that the phosphorescence emission peaks ranged from 520 nm to 570 nm, and their emission peaks were similar to those of DDF-O. This suggested that the guest molecule’s triplet exciton generated phosphorescence in the doped system.

The phosphorescence emission peaks of the two host materials, TPP and 2MoBPA, were similar. However, their afterglow colors differed, possibly caused by the different photo-physical processes after combining the host and guest. To gain an improved understanding of the mechanisms through which electron-donating capacity regulates energy arrangement and orbital characteristics, HOMO–LUMO levels, electrostatic potential distribution, electron transfer between the host and guest, and energy-level distributions are calculated by Density Functional Theory (Figure S2). The LUMO values of the TPP_2_: DDF-O, 2MoBPA: DDF-O, TPA: DDF-O, and CHPL: DDF-O systems are distributed on the guest, while the HOMO values are distributed on the host, allowing electron transition between the guest and host to form electron transfer. The electron transfer between the host and guest was calculated, showing that the TPP_2_: DDF-O transferred electrons have an energy value 0.009 eV. We explored the photo-physical properties of TPP_2_: DDF-O and 2MoBPA: DDF-O. To obtain the highest occupied molecular orbital (HOMO) energy level *E*_HOMO_ and the lowest unoccupied molecular orbital (LUMO) energy level *E*_LUMO_ of the DDF-O, 2MoBPA, and TPP monomers, we used the BAS 100W electrochemical analyzer to test their cyclic voltammetry (CV) curves, as shown in Table S1 and Figures S3 and S4. Simple analysis located the HOMO energy levels of DDF2o, 2MoBPA, and TPP_2_ at −5.1, −5.5, and −5.8 eV, respectively. The LUMO energy levels of DDF-O, 2MoBPA, and TPP were calculated from the formula *E*_g_ = *E*_HOMO_ − *E*_LUMO_ as −2.1, −2.3, and −2.4 eV, respectively. The test results indicated that the LUMO energy level of DDF-O was higher than those of 2MoBPA and TPP. Therefore, DDF-O could act as the donor, while 2MoBPA and TPP could serve as the acceptor, allowing their energy levels to match and form a donor–acceptor (D–A) system. The charge transfer between host and guest was the key to realizing RTP.

**Figure 4:**
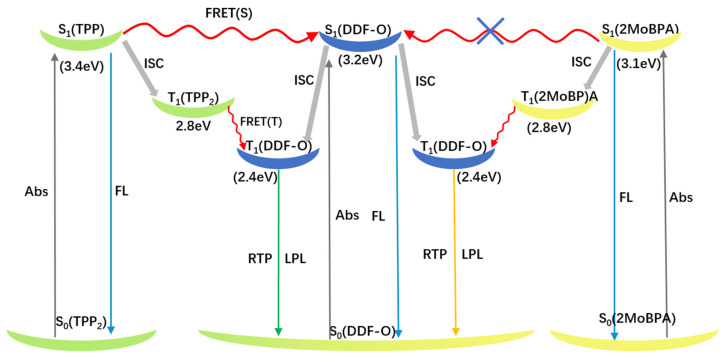
Förster resonance energy transfer (FRET) processes of doped crystals TPP: DDF-O and 2MoBPA: DDF-O upon 365 nm excitation.

The rigid environment provided by the hosts is necessary for the guest/host system to display RTP characteristics. However, is this the only role the host molecules play? Energy transfers between host and guest molecules have been revealed to play a vital role in phosphorescence activity. We therefore speculated that Förster resonance energy transfer (FRET) was a viable explanation (Figure 4).

The energy levels of the singlet and triplet states of DDF-O, TPP_2_, and 2MoBPA are summarized in Table S1. The singlet energy level of DDF-O (3.2 eV) was higher than that of 2MoBPA (3.1 eV) and lower than that of TPP_2_ (3.4 eV). Therefore, the Förster energy transfer process could occur between the singlet energy level of DDF-O and that of TPP_2_ in the doped system. After the excitation of the host material TPP_2_, the generated singlet exciton energy could be transferred to the guest material DDF-O and then decayed by the fluorescence emission pathway, enhancing DDF-O’s fluorescence effect. The fluorescence decay curves further supported this conclusion. The fluorescence emission decay of TPP_2_: DDF-O at 420 nm fitted a single exponential curve with a lifetime of 1.05 ns, indicating that energy transfer between the host and guest nearly caused the TPP_2_ fluorescence to disappear and enhanced the fluorescence emission of DDF-O. In contrast, since the singlet energy level of DDF-O was higher than that of 2MoBPA and no Förster energy transfer could occur between the singlet states of DDF-O and 2MoBPA, the fluorescence decay of 2MoBPA: DDF-O fitted a bi-exponential curve, with lifetimes of 1.97 ns and 0.79 ns corresponding to the fluorescence emission decay of 2MoBPA and DDF-O, respectively. As shown in the figure, the phosphorescence peak at 446 nm for TPP:DDF-O was significantly weaker than that of pure TPP, while the phosphorescence peak at 560 nm was markedly enhanced, indicating an energy transfer process between the triplet energy levels of the host and guest. The energy was transferred from the triplet energy level T1 (2.8 eV) of TPP to T1 (2.4 eV) of DDF-O, where the triplet exciton from the T1 energy level of DDF-O then relaxed to the ground state. The long-afterglow generation mechanism of 2MoBPA: DDF-O was similar to that of TPP:DDF-O. The phosphorescence peak at 450 nm of 2MoBPA: DDF-O was significantly weaker than that of pure 2MoBPA, while the phosphorescence peak at 520 nm was dramatically enhanced, and the triplet energy transferred from the 2MoBPA triplet energy level to DDF-O.

The Förster energy transfer process occurred between DDF-O and TPP, while no energy was transferred between DDF-O and 2MoBPA, resulting in different afterglow colors. The afterglow duration difference was directly related to the phosphorescence lifetime. The phosphorescence emission decay spectra showed that the phosphorescence lifetime (*τ*) of 2MoBPA: DDF-O was 290.21 ms, while that of TPP_2_: DDF-O was 383.16 ms (Figure 3). The efficient RTP lifetime led to the difference in afterglow duration of the doped materials.

**Figure 5:**
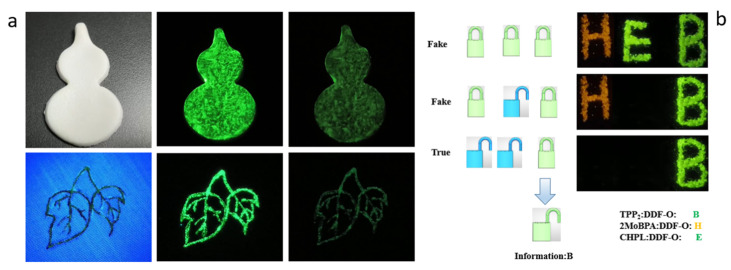
(**a**) LPL photograph of copolymer-doped crystals 4N-TCD: DPA-PVA-H under ambient conditions, excited with 365 nm light at various temperatures. (**b**) Illustration of multiple information encryption of “EB” letters, based on the doped materials of 4N-TCD: DPA and MODPA: DDF-O. (**b**) 4N-TCD: DPA: PVP paint applied to different substrates.

Figure 5 illustrates the application of time- and color-tunable host/guest doping systems in information anti-counterfeiting display and security protection. As illustrated in Figure 5b, the materials TPP_2_: DDF-O, 2MoBPA: DDF-O, and CHPL: DDF-O were used to create the abbreviation “HEB”, representing Hebei Petroleum University of Technology. Under the excitation of a 365 nm UV lamp, these materials activate their respective signature locks. When the UV lamp is turned off, 2MoBPA:DDF-O exhibits the “H” lock, CHPL: DDF-O exhibits the “E” lock, and TPP_2_: DDF-O exhibits the “B” lock. Owing to its longer afterglow lifetime, the TPP_2_: DDF-O material produces the most persistent afterglow. Thus, the final visible message is “B,” which enhances the security of the encrypted information. All the films were produced by screen printing technology, and the doped material was printed on various substrates. Good afterglow performance was demonstrated on substrate surfaces such as paper, clothing, and gypsum, indicating that such materials could be widely used in different anti-counterfeiting applications (Figure 5a).

## 3. Materials

DDF-O (C_41_H_36_N_2_O_2_), TPP_2_ (C_30_H_24_P_2_), Triphenylamine (TPA) (C_18_H_15_N), 2MoBPA (C_15_H_14_O_3_), and 1-cyclohexyl-3-phenylthiourea (CHPL) (C_13_H_18_N_2_S) were purchased from Tianjin Xiensi Biochemical Technology Co., Ltd. (Tianjin, China), and above compounds were further purified by recrystallization (purity: ≥99.9%). PVP (average molecular weight: 58,000) was purchased from Tianjin Heowns Biochemical Technology Co., Ltd. (Tianjin, China).

Preparation of trace-doped crystal TPP_2_: DDF-O. At room temperature, 5 mL of anhydrous ethanol and 0.5 mL dichloromethane were added to 1%TPP_2_: 99 mol% DDF-O mixed powder (0.50 g), and the solution was prepared using an ultrasonic cleaner to dissolve and uniformly mix the donor and acceptor materials. Evaporates at room temperature, yielding a white crystalline product.

Preparation of trace-doped crystal TPA: DDF-O, 2MoBPA: DDF-O, and CHPL: DDF-O. The preparation process of TPA: DDF-O, 2MoBPA: DDF-O, and CHPL: DDF-O was similar to that of TPP_2_:DDF-O, which only replaced the TPP_2_ in the raw material with TPA, 2MoBPA, and CHPL, respectively.

Preparation of the TPP_2_: DDF-O: PVP paint: 1.0 g of the trace-doped (5 mol‰) TPP_2_: DDF-O powder and 3.0 g of PVP were added into dichloromethane (8.0 mL), and then the mixture was stirred at room temperature to produce the paint in which the mass percentage of host/guest doping material was 25 wt% (without solvent).

### Measurement and Characterization

The room-temperature photoluminescence (PL), phosphorescence, and LPL spectra of the crystals were recorded with an Ocean Optics fiber spectrophotometer (Dunedin, NZ, USA). Ultraviolet-visible (UV–vis) spectroscopy was performed using a Thermo Spectronic Helios Gamma spectrometer (Thermo Electron North America, West Palm Beach, FL, USA). Quartz cells had a path length of 1 cm. Fluorescence (FL) spectroscopy was carried out using a Varian CARY ECLIPSE fluorescence spectrometer (Varian Australia Pty Ltd., Mulgrave, VIC, Australia). Time-resolved FL and PL experiments were performed with a spectrophotometer (Gilden Photonics, Clydebank, UK) using a pulsed source at 480 nm (BDS-SM ps diode laser, Becker & Hickl GmbH, Berlin, Germany). The time-resolved signals were recorded by a time-correlated single-photon counting detection technique. SEM images were recorded via a Hitachi JSM-7800F field emission microscope (Tokyo, Japan) equipped with an EDX spectrometer [33]. Single-crystal X-ray diffraction was performed using a SuperNova diffractometer (Agilent, Wrocław, Poland), which had a CCD detector and rotating-anode X-ray generator [34].

## 4. Conclusions

A series of host/guest doping long-afterglow materials with superior RTP performance were prepared in this study. A transition from yellow to green afterglow was achieved by modifying the molecular structure of the host. The phosphorescence lifetime could be adjusted over a wide range, from 290.21 ms to 383.15 ms, and the stable afterglow luminescence could be maintained for 1–4 s under ambient conditions. The rigid structure of the host molecule, the appropriate triplet energy level, the Förster energy transfer from the host triplet state to the guest triplet state, and the efficient ISC process were the keys to the efficient RTP and long afterglow of the doped materials. Based on this wide adjustable time and color range of the host/guest doping RTP system, we could easily realize complex and high-level information encryption and multi-dimensional dynamic anti-counterfeiting, leveraging the coding features and temperature stimulus response diversity. In particular, the flexible afterglow properties of these RTP materials hold promise for applications in wearable electronic devices.

## Data Availability

The original contributions presented in this study are included in the article/Supplementary Materials. Further inquiries can be directed to the corresponding author(s).

## Supplementary Materials

The following supporting information can be downloaded at https://www.mdpi.com/article/10.3390/molecules30061203/s1, Figure S1: XRD spectra of the doped crystals: (a) TPP_2_: DDF-O, (b) 2MoBPA: DDF-O, (c) TPA: DDF-O, and (d) CHPL: DDF-O; Figure S2: Calculation of ground and excited state geometry, HOMO-LUMO energy level distribution, electron transfer between host and guest, and electrostatic potential using DMol3 program (calculation methods in Supporting Information Characterization data).; Figure S3: Electrochemical curves of TPP_2_ in dichloromethane vs. Ag/Ag^+^, with the concentration of 5 × 10^−3^ mol·L^−1^.; Figure S4: UV–vis absorption (black) spectra, fluorescence (red), and phosphorescence (blue) of the TPP_2_ (excitation: 365 nm), measured at room temperature.; Table S1: Frontier orbital energies (HOMO and LUMO) and some energy level data upon excitation for DDF-O, TPP_2_, and 2MoBPA. References [35,36,37,38,39,40,41] are cited in the Supplementary Materials.
