# Supplementary material for: Multi-Color Tunable Afterglow Materials Leveraging Energy Transfer Between Host and Guest
Source: Molecules. 2025 Mar 7;30(6):1203. doi: 10.3390/molecules30061203 (PMC11945158; doi:10.3390/molecules30061203)
Supplement: Supplementary file 1 [file molecules-30-01203-s001.zip › molecules-3433134-supplementary.pdf]

Supporting Information

Multi-color tunable afterglow materials leveraging energy transfer between host and guest

Xiao He[1], Bo Wang[1*], Xiaoqiang Zhao[1**], Fengqin Ke[1], Wenhui Feng, Liwen Wang[1], Jiameng Yang[1], Guangyu Wen[2], and Denghui Ji[3***]

[1] Hebei Petroleum University of Technology, Cheng De City 067000, P. R. China;

[2]College of Physics and Hebei Advanced Thin Film Laboratory, Hebei Normal University, Shijiazhuang City, 050024, P. R. China;

[3]Science College, Shijiazhuang University, Shijiazhuang City 050035, P. R. China.

[*]E-mail: wb_55428335@126.com [**]E-mail: zxq130823@163.com

[***]E-mail: jidenghui2007@163.com

Table of contents

1..........Characterization data S2

2.......... Supplementary tables and figures S3

3..........References S7

1. Characterization data

TC-SPC. Sample excitation was conducted with picosecond diode lasers (Horiba Jobin Yvon Instruments) at 321 nm or 375 nm, and the time resolution was ~ 150 ps. The laser pulse energy was ca. 15 pJ and attenuated (often by more than an order of magnitude) to the desired count rate of ca. 1% or less of the excitation frequency. A cooled (ca. -40°C) Hamamatsu MCP- photomultiplier R3809U 51 was used for detection of single photons, and the signal passed through a discriminator (Ortec 9307) and into a TAC (Ortec 566, 100 ns range used). The electrical trigger signal from the laser was also passed through a discriminator (Tennelec TC454) and on to the TAC (Ortec 566). The TAC output was read by a DAQ-1 MCA computer card using 1024 channels and collected with Horiba Jobin Yvon Data Station 2.5. Measurements were conducted in reverse mode at 5 MHz and under magic angle polarization. A cut-off filter, GG400 (Excitation at 321 nm) or GG 515 (Excitation at 375 nm), was used to block stray excitation light. A dilute solution of Ludox was used to record the instrument response function without any filter for solution measurements. No monochromator was used; i.e., all wavelengths transmitted by the cut-off filter were collected.

Computational Methods

Utilizing the density functional theory, the calculations were executed through the DMol3 program[1-3]. The exchange-correlation functional employed was the generalized gradient approximation developed by Perdew, Burke, and Ernzerhof [4]. This computational method mirrors the methodology used in our previous investigation of the interaction between polyacrylamide and H_2O[5]. The basis set selected was Double Numerical plus Polarization, with the basis file defaulting to version 4.4. DFT semi-core pseudopotentials were configured as DFT-based potentials, and spin polarization was set to unrestricted. The quality of the global orbital cut-off was refined to fine, with a value of 3.7 Å, and a Fermi smearing of 0.005 Ha was applied. Additionally, we imposed the following convergence criteria: an energy tolerance of 1.0×10^{-5} Ha/atom, a self-consistent field tolerance of 1.0×10^{-6} Ha/atom, a maximum force tolerance of 0.002 Ha/Å, and a maximum displacement tolerance of 0.005 Å.

2. Supplementary table and figures

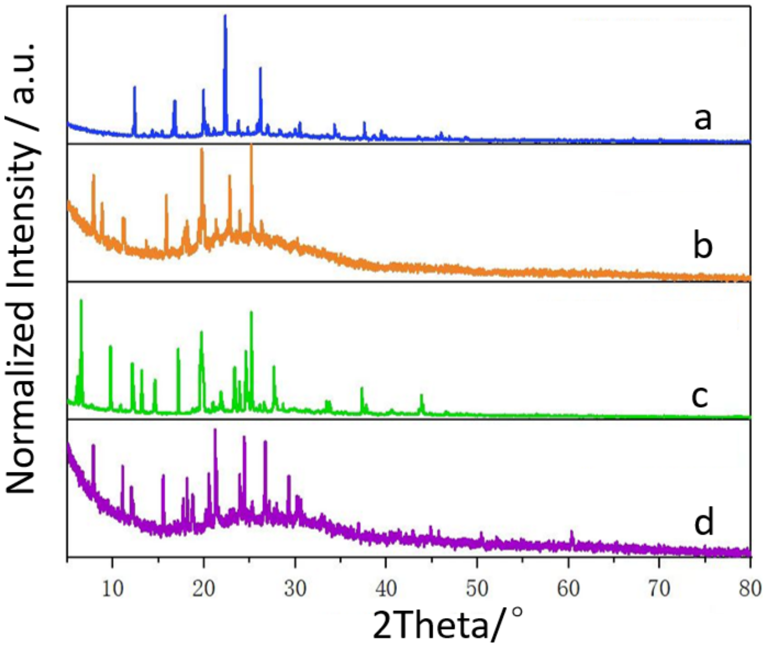

Figure S1. XRD spectra of the doped crystals: (a) TPP_2: DDF-O, (b) 2MoBPA: DDF-O, (c) TPA: DDF-O, and (d) CHPL: DDF-O.

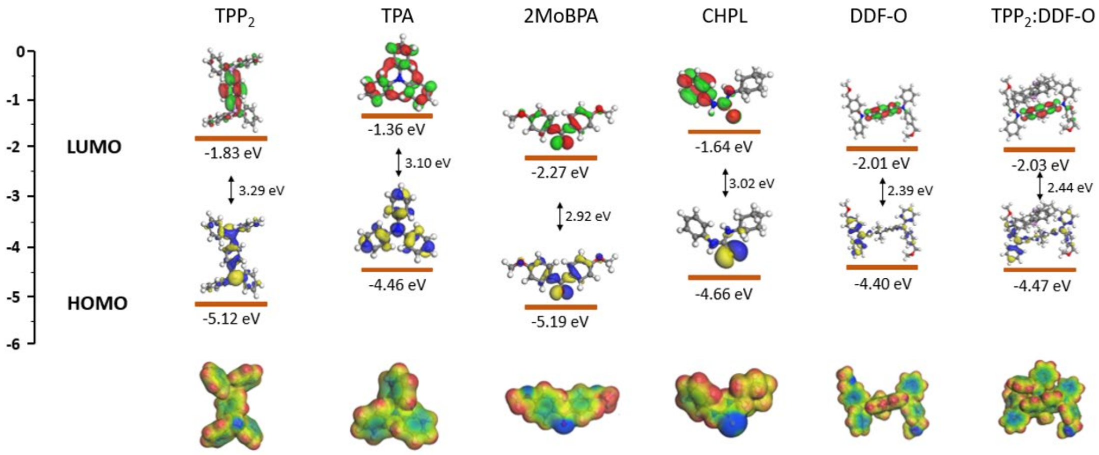

Figure S2. Calculation of ground and excited state geometry, HOMO-LUMO energy level distribution, electron transfer between host and guest, and electrostatic potential using DMol3 program (calculation methods in Supporting Information Characterization data).

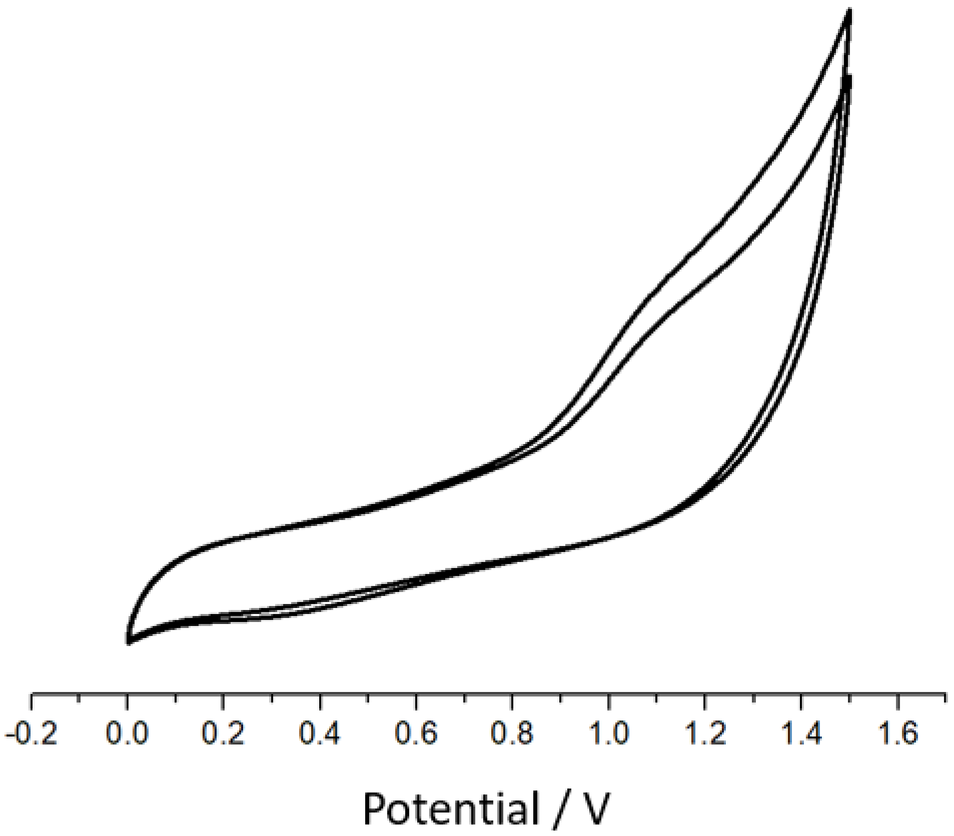

Fig.S1

Figure S3. Electrochemical curves of TPP_2 in dichloromethane vs. Ag/Ag^+, with the concentration of 5×10^{-3} $mol{\bullet}L^{-1}$.

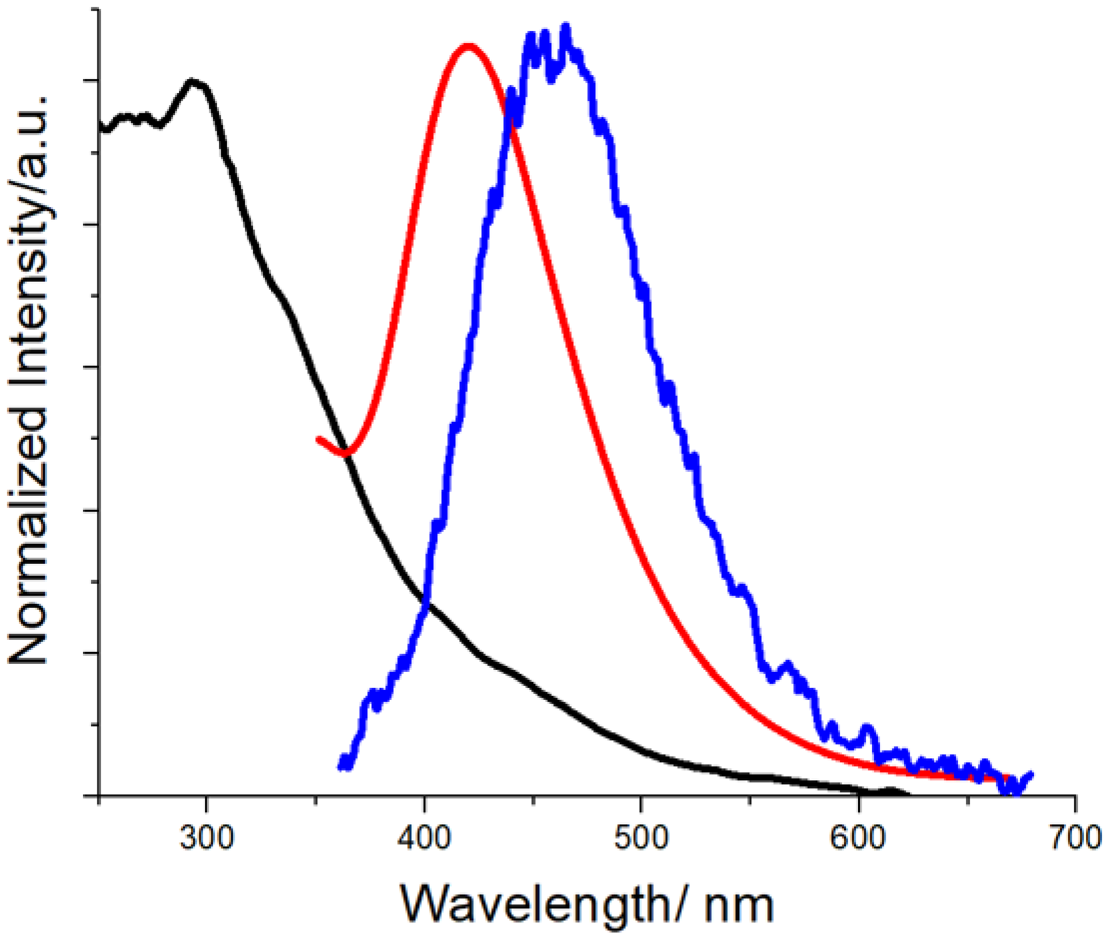

Figure S4. UV–vis absorption (black) spectra, fluorescence (red), and phosphorescence (blue) of the TPP_2 (excitation: 365 nm), measured at room temperature.

Table S1. Frontier orbital energies (HOMO and LUMO) and some energy level data upon excitation for DDF-O, TPP_2, and 2MoBPA.

Sample	HOMO[a)] (eV)	LUMO[b)] (eV)	λ_{max}(abs) (nm)	λ_{max}(fl) (nm)	$E(S_1)$[c)] (eV)	λ_{max}(ph) (nm)	$E(T_1)$[d)] (eV)
DDF-O[6]	-5.1	-2.1	382	400	3.2	515	2.4
TPP_2	-5.8	-2.4	307	430	3.4	446	2.8
2MoBPA[7]	-5.5	-2.3	356	448	3.1	540	2.8

a) The HOMO energy was deduced from the oxidation onset potential from cyclic voltammetry data and calculated by the equation $E_{HOMO} = -E_{onset} - 4.93$ (eV).

b) $E_{LUMO} = E_{HOMO} + Eg_{op}$, the optical band gap ($Eg_{op}^{Host}$, the values were 3.4 eV and 3.2 eV for crystals TPP_2 and 2MoBPA, respectively; Eg_{op}^{Guest}, the value was 3.0 eV for crystal DDF-O), was estimated from the onset of the absorption band.

c) Singlet energies $E(S_1)$ were calculated from absorption and fluorescence spectra of the crystals of DDF-O, TPP2, and 2MoBPA; that is, $E(S_1) = 1241/[(\lambda_{abs}+\lambda_{fl})/2]$.

d) Triplet energies $E(T_1)$ were due to phosphorescence spectra of the DDF-O, TPP2, and 2MoBPA crystals with the formula $E(T_1) = 1241/\lambda_{ph.}$

References

[1] B. Delley, J. Chem. Phys. 1990, 92, 508.

[2] B. Delley, J. Phys. Chem. 1996, 100, 6107.

[3] B. Delley, J. Chem. Phys. 2000, 113, 7756.

[4]Perdew J P, Burke K, Ernzerhof M. Generalized gradient approximation made simple[J]. Physical review letters, 1996, 77(18): 3865.

[5] Wei T, Ren Y, Li Z, et al. Bonding interaction regulation in hydrogel electrolyte enable dendrite-free aqueous zinc-ion batteries from −20 to 60°C[J]. Chemical Engineering Journal, 2022, 434: 134646.

[6] D. Y. Muleta, J. Song, W. Feng, R. Wu, X. Zhou, W. Li, L. Wang, D. Liu, T. Wang and W. Hu. Small molecule-doped organic crystals towards long-persistent luminescence in water and air. J. Mater. Chem. C, 2021, 9, 5093.

[7] J. Mao， W. Feng, T. Wang. Effect of Guest Isomerism on Afterglow Color of Organic Host-guest Crystals. Chem. J. Chinese Universities, 2023, 44(6), 20220771.
